# Extending research on Emotion Regulation Individual Therapy for Adolescents (ERITA) with nonsuicidal self-injury disorder: open pilot trial and mediation analysis of a novel online version

**DOI:** 10.1186/s12888-018-1885-6

**Published:** 2018-10-11

**Authors:** Johan Bjureberg, Hanna Sahlin, Erik Hedman-Lagerlöf, Kim L. Gratz, Matthew T. Tull, Jussi Jokinen, Clara Hellner, Brjánn Ljótsson

**Affiliations:** 1 Centre for Psychiatry Research, Department of Clinical Neuroscience, Karolinska Institutet, & Stockholm Health Care Services, Stockholm County Council, Norra stationsgatan 69, SE-11364 Stockholm, Sweden; 2 Division of Psychology, Department of Clinical Neuroscience, Karolinska Institutet, Nobels väg 9, SE-171 65 Stockholm, Sweden; 3 Department of Psychology, University of Toledo, 2801 W. Bancroft Street, Toledo, OH 43606 USA; 4 Department of Clinical Sciences/Psychiatry, Umeå University, By 23, Enheten för psykiatri, 901 85 Umeå, Sweden

**Keywords:** Nonsuicidal self-injury disorder, Self-injurious behavior, Emotion regulation individual therapy, Emotion regulation, Online treatment, Internet-based treatment

## Abstract

**Background:**

Nonsuicidal self-injury (NSSI) is common among adolescents and associated with negative outcomes. However, treatments developed specifically for NSSI and the proposed NSSI disorder (NSSID) are scarce, and access to empirically supported treatments for NSSI in many areas is limited. Online treatments carry the potential to increase the availability of evidence-based treatments. Emotion regulation individual therapy for adolescents (ERITA) has shown promise in the treatment of adolescents with NSSID.

**Method:**

The present study examined the feasibility, acceptability, and utility of an online version of ERITA. Twenty-five adolescents (aged 13–17) with NSSID and their parents were included in an uncontrolled open trial. Self-report and clinician-rated assessments of outcomes such as NSSI, self-destructive behaviors, emotion dysregulation, and global functioning were administered at pre-treatment, post-treatment, 3- and 6- month follow-up. Measures of NSSI, self-destructive behaviors, and emotion dysregulation were also assessed weekly during treatment.

**Results:**

Ratings of treatment credibility, expectancy, and satisfaction were acceptable, and the therapeutic alliance and treatment completion rate (96%) were high. Adolescent participation in the treatment was associated with a statistically significant increase in past-month NSSI abstinence (*p =* .007), large-sized improvements in past-month NSSI frequency (55% reduction, 95% confidence interval [CI]: 29, 72; Cohen’s *d* = 0.88, 95% CI: 0.73, 1.06) and global functioning (*d* = 1.01, 95% CI: 0.77, 1.32), and medium-sized improvements in emotion dysregulation (*d* = 0.75, 95% CI: 0.59, 0.90) and NSSI versatility (*d* = 0.63, 95% CI: 0.54, 0.77) from pre- to post-treatment. These improvements were further strengthened at 3-month follow-up and maintained at 6-month follow-up. The online therapist-guided parent program was associated with small- to large-sized (*ds =* 0.47–1.22) improvements in adaptive parent behaviors, and these improvements were maintained or further improved upon at 6-month follow-up. Moreover, in line with the theoretical model underlying ERITA, change in emotion dysregulation mediated changes in both NSSI frequency and self-destructive behaviors over the course of treatment.

**Conclusions:**

Together, results suggest that online ERITA is an acceptable, feasible, and promising low-intensity treatment for adolescents with NSSID. The results of this open trial must be replicated in controlled studies.

**Trial registration:**

ClinicalTrials.gov (NCT02697019). Registered 2 March 2016

**Electronic supplementary material:**

The online version of this article (10.1186/s12888-018-1885-6) contains supplementary material, which is available to authorized users.

## Background

Nonsuicidal self-injury (NSSI) is defined as the deliberate self-inflicted destruction of body tissue without suicidal intent and for purposes not socially sanctioned [1]. NSSI disorder (NSSID) was introduced in the most recent version of the Diagnostic and Statistical Manual of Mental Disorders (DSM-5 [2]) as a disorder for further study. NSSID has a prevalence of 3.1 to 6.7% in adolescent community samples [3, 4], and both NSSI and NSSID have been associated with a variety of negative outcomes, including general psychopathology and suicide attempts [5, 6]. Despite the potentially severe consequences of NSSI and NSSID, there are few publications on interventions specifically designed to treat NSSI [7–10]. Moreover, although a recent review of treatments for suicidal and nonsuicidal self-injurious behaviors among adolescents [9] identified three treatments as promising (including dialectical behavior therapy [11, 12], mentalization-based treatment [13], and cognitive behavior therapy [CBT]; [14, 15]), the authors concluded that no treatment for NSSI in adolescents (when analyzed separately from suicide attempts) is superior to treatment as usual.

To address the relative lack of effective treatments for youth with NSSI, we developed emotion regulation individual therapy for adolescents (ERITA [16]). ERITA is a 12-week, acceptance-based behavioral individual therapy adapted from emotion regulation group therapy (ERGT) for NSSI in adults [17, 18]. Similar to ERGT, ERITA was developed specifically to decrease NSSI among adolescents by improving emotion regulation skills. In the initial pilot study of ERITA, this treatment was delivered to adolescents in a traditional face-to-face format. The parents also participated in a parallel online parent program developed to increase their ability to interact effectively with their adolescents to decrease their child’s NSSI. The utility, acceptability, and feasibility of face-to-face ERITA for NSSID was supported in the open pilot trial including 17 adolescents (aged 13–17 years) and their parents [16]. Participants rated the treatment as credible, and both the treatment completion rate (88%) and treatment attendance were high. Intent-to-treat analyses revealed large-sized uncontrolled effects from pre- to post-treatment in past-month NSSI frequency, emotion dysregulation, self-destructive behaviors, and global functioning, as well as a medium-sized effect in past-month NSSI versatility. All of these improvements were either maintained or further improved upon at 6-month follow-up. Moreover, change in emotion dysregulation mediated the observed improvements in NSSI during treatment, providing preliminary support for the theoretical model underlying ERITA [16].

ERITA was developed to provide a targeted and effective intervention that could be easily and widely implemented at a low cost. However, Internet-delivered CBT (ICBT) has the potential to further increase accessibility to evidence-based treatments by eliminating the effects of geographical distances between patients and providers, allowing for less therapist time per patient, and facilitating flexible scheduling at times that are convenient for families [19]. Therapist-guided ICBT has been shown to be effective for several psychiatric and physical disorders in adolescents (for a review, see [20]). There is also research indicating that ICBT is at least as efficacious as face-to-face CBT for a range of psychiatric disorders in adults [21]. Further, given research indicating that individuals with stigmatizing illnesses are more likely to use the Internet than traditional health care services to seek help [22], online treatment may be particularly suitable for the treatment of NSSI, given its association with shame [23] and low levels of disclosure [24]. Nonetheless, although there have been some efforts to develop web-based interventions for suicidal behaviors (e.g. [25]), online interventions have not (to our knowledge) been evaluated specifically for individuals who engage in NSSI.

Thus, given both evidence for the utility and feasibility of ERITA delivered face-to-face and the advantages of the ICBT format, we adapted ERITA to an online intervention. Following recommendations for early research on novel interventions [26, 27], the present pilot study examined the feasibility, acceptability, and utility of this online ERITA in an uncontrolled open pilot trial. We expected high levels of treatment module completion, low treatment attrition, and high treatment credibility and satisfaction. Further, we expected to find significant improvements from pre- to post-treatment in adolescent NSSI, emotion regulation difficulties, psychological inflexibility, global functioning, and symptoms of borderline personality disorder (BPD), as well as parents’ ability to respond effectively to their children’s negative emotions. We also anticipated that these improvements would be maintained or further improved upon at 3- and 6-month follow up periods. Finally, we hypothesized that change in emotion dysregulation would mediate improvements in NSSI and self-destructive behaviors during treatment.

## Method

### Design

This study employed an uncontrolled open trial design evaluating online ERITA for NSSID among adolescent participants in Stockholm, Sweden. Participants were either self-referred or referred from child and adolescent mental health services (CAMHS). Referring clinics received information about the trial through written information distributed via emails and the Stockholm County Council’s internal website. Self-referred participants were recruited through advertisements in the local newspaper and through information posted on social media and the Swedish National Self-Injury Project’s website. The adolescent treatment included 11 online modules adapted from the ERITA manual [16]. As in the previous ERITA trial [16], parents were enrolled in a parent program also delivered via the Internet. Demographic, diagnostic, and baseline data were collected through self-report measures and via interviews conducted by licensed psychologists. Self-report measures were also administered weekly beginning 2 weeks prior to treatment and continuing throughout treatment. Post-treatment and follow-up assessments were administered directly after treatment completion (i.e., 11 weeks after treatment start) and at 3 and 6 months post-treatment completion. These included both clinician administered interviews and self-report measures. The self-report measures used in the study were administered online which has been shown to be a reliable and valid method [28].

The study was registered on Clinicaltrials.gov (Identifier NCT02697019), and the TREND Statement guidelines for nonrandomized interventions [29] were followed when reporting the trial.

### Participants

**Fig. 1:**
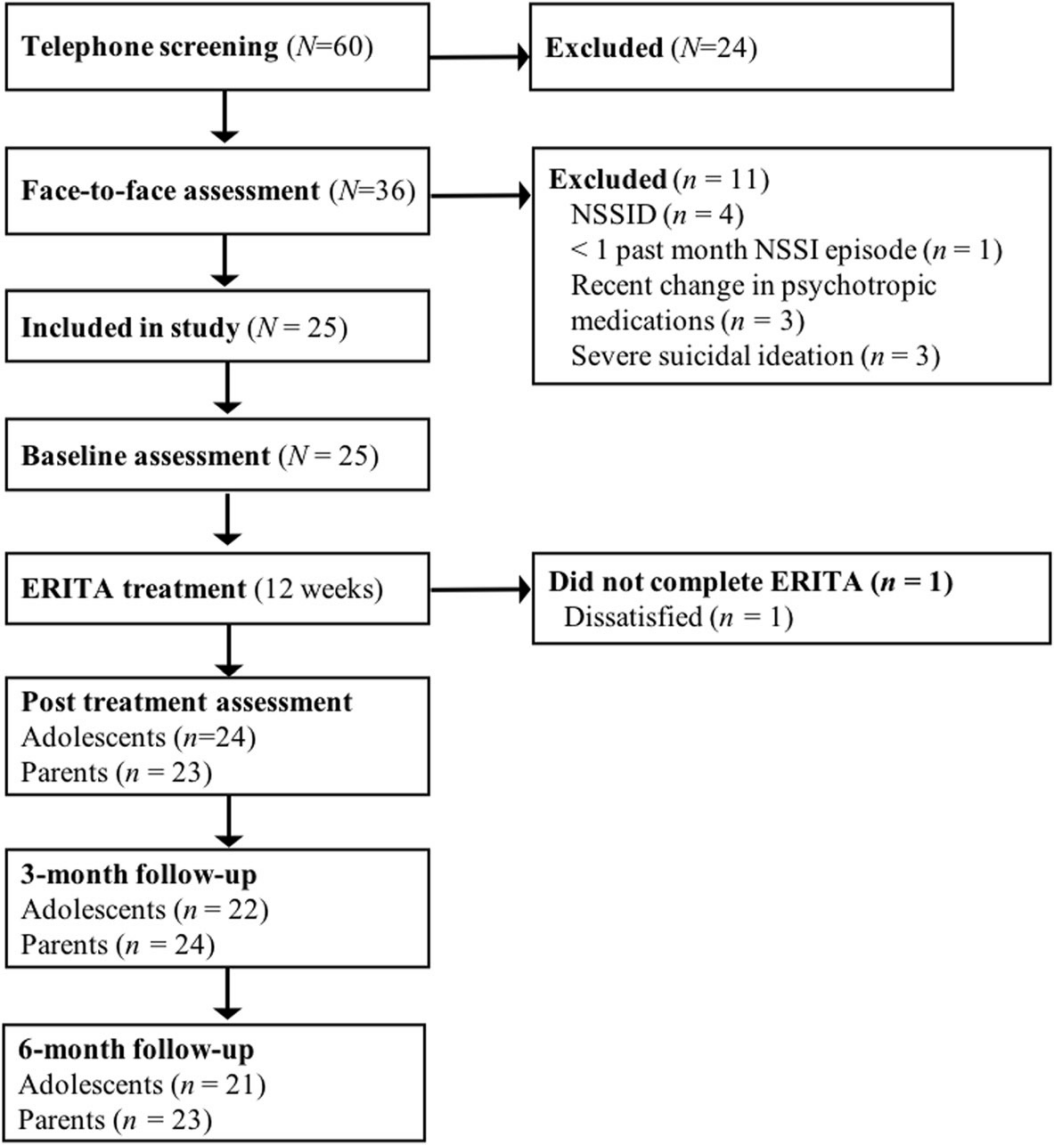
Participant flow through the study

A total of 60 families were screened by telephone for eligibility between March 2016 and March 2017. Thirty-six of these families were assessed face-to-face, and 25 met inclusion criteria. Of these, 19 (76%) were self-referred and six (24%) were referred from CAMHS. The inclusion criteria for the adolescents were: (a) 13–17 years of age; (b) meeting diagnostic criteria for NSSID [2]; (c) having engaged in ≥1 NSSI episode during the past month; (d) having at least one parent who committed to participate in the parent program; and (e) stability of psychotropic medications (if any) for at least 2 months. Exclusion criteria for the adolescents were: (f) severe suicidal ideation; (g) a diagnosis of psychotic or bipolar I disorder or ongoing (past month) substance dependence; (h) ongoing dialectical behavioral therapy or mentalization-based treatment; and (i) insufficient understanding of the Swedish language. Of the enrolled participants, 92% met diagnostic criteria for at least one psychiatric disorder other than NSSID, and the median length of previous psychological treatment was 9 months. Demographic and diagnostic data are described in Table 1, and participant flow through the trial is presented in Fig. 1.

**Table 1:** Sociodemographic, clinical, and diagnostic data of the sample (*N* = 25)

Age (years)	*M (SD)*	15.7 (1.3)	
	Min-max	13.4–17.7	
Gender	Female	19	76%
	Male	1	4%
	Other (non-binary)	5	20%
Country of birth	Sweden	24	96%
	Other European country	1	4%
Gender of treatment responsible parent	Female	22	88%
	Male	3	12%
Treatment responsible parent’s educational level	Primary	1	4%
	Secondary	7	28%
	University	17	68%
Ongoing psychotropic medication	Yes	8	32%
Earlier psychological treatments	Yes	15	60%
	Mean length in months (*SD)*	11.9 (16.4)	
Meeting full diagnostic criteria for BPD	Yes	5	20%
Mean number of BPD criteria		3.2 (1.6)	
Adolescent’s report on age of NSSI onset	*M (SD)*	13.3 (1.3)	
Parent’s report on age of their child’s NSSI onset	*M (SD)*	14.1 (1.6)	
NSSI frequency past 12 months	Median	103	
	IQR	25–197	
Frequency of co-occurring disorders	Depression	13	52%
	Panic disorder	9	36%
	ADHD	3	12%
	Social anxiety disorder	13	52%
	Separation anxiety	1	4%
	Specific phobia	2	8%
	Bulimia nervosa	1	4%
	GAD	7	28%
	BDD	5	20%
Number of participants with 0 - ≥5 co-occurring disorders	None	2	8%
	One	3	12%
	Two	9	36%
	Three	5	20%
	Four	2	8%
	≥Five	5	16%

*Note. ADHD* Attention deficit hyperactivity disorder, *BDD* Body dysmorphic disorder, *BPD* Borderline personality, *GAD* Generalized anxiety disorder, *IQR* Interquartile range, *NSSI* Nonsuicidal self-injury

### Measures

#### Diagnostic assessments

A full diagnostic interview was conducted using the MINI-KID International Neuropsychiatric Interview, version 6 [30]. The Clinician-Administered Nonsuicidal Self-Injury Disorder Index [31] was used to determine if participants met criteria for NSSID, and the Structured Clinical Interview for DSM-IV Personality Disorders BPD Module [32] was used to assess diagnostic criteria for BPD.

#### Adolescent-rated outcome measures

##### Acceptability measures

Perceived credibility and expectancy of the treatment was measured using the Credibility/Expectancy Scales [33]. Higher scores indicate greater credibility (range 1–9) and expectancy (range 0–100%). Evidence for the reliability and predictive validity of this measure has been provided [34]. This measure was administered after the first treatment module. Satisfaction with the treatment was measured using the self-report measure, Client Satisfaction Questionnaire – 8 item version [35]. Scores on this measure range from 8 and 32, with higher scores indicating greater treatment satisfaction. Internal consistency of this measure in the adolescent sample was excellent (α = .92). Aspects of therapeutic alliance were measured using the 12-item version of the Working Alliance Inventory – Revised Short Version [36], which has demonstrated sound psychometric properties [36]. Scores range between 12 and 84, with higher scores indicating better therapeutic alliance. This measure was administered after the third treatment module, and internal consistency in the present sample was excellent (α = .95). At the post-treatment assessment, participants completed a self-report measure of the occurrence of any potential adverse events associated with the online intervention.

##### Primary outcome measure

The primary outcome of NSSI frequency was measured using the 9-item Deliberate Self Harm Inventory (DSHI-9 [37]). The DSHI-9 assesses the presence and frequency of the most common forms of NSSI, including cutting, burning, severe scratching, self-biting, carving, sticking sharp objects into the skin, self-punching, and head banging. For this study (and consistent with the pilot study on ERITA [16]), the item assessing presence and frequency of preventing wounds from healing was not included, as this behavior is considered to be relatively normative and a less severe form of self-injury. Evidence for the test-retest reliability and concurrent validity of the DSHI-9 has been provided among adolescents [37]. The DSHI-9 was also used to assess past month NSSI versatility (i.e., the number of different types of NSSI behaviors), which has been shown to be a marker of NSSI severity [38]. The DSHI-9 was administered at pre-treatment, post-treatment, and 3- and 6-month follow-up to assess past month engagement in NSSI. The DSHI-9 was also administered weekly during treatment to measure past week engagement in NSSI.

##### Secondary outcome measures

Participants’ emotion regulation difficulties were assessed using the 36-item Difficulties in Emotion Regulation Scale (DERS [39]). Scores on this scale range from 36 to 180, with higher scores indicating greater difficulties in emotion regulation. The DERS has demonstrated good reliability and construct and convergent validity in adolescents [40]*.* The DERS was administered at pre-treatment, post-treatment, and 3- and 6-month follow-up. Internal consistency in this sample was excellent (α = .90). Emotion regulation difficulties were also measured weekly during treatment using the 16-item version of the DERS, the DERS-16 [41]. Scores on this scale range from 16 to 80, with higher scores indicating greater emotion regulation difficulties. The DERS-16 has been found to demonstrate good test-retest reliability and construct and predictive validity among adults [41–43]. Internal consistency in this sample was good (α = .89).

Past week engagement in a variety of risky, self-destructive behaviors (e.g., risky sexual behavior, binge eating, substance misuse) was measured using the 11-item behavior supplement to the Borderline Symptom List (BSL [44]). Scores on this measure range from 0 to 55. The BSL-supplement was administered weekly during treatment. BPD features were measured using the Borderline Personality Feature Scale for Children (BPFS-C [45]). The BPFS-C is a 24–item self-report measure with scores ranging from 24 to 120; higher scores indicate greater BPD features. The BPFS-C has demonstrated adequate construct validity among youth [45]. The BPFSC was administered at pre-treatment, post-treatment, and 3- and 6-month follow-up. Internal consistency in this sample was good (α = .84). Finally, psychological inflexibility was measured using the 7-item Acceptance and Action Questionnaire (AAQ-II [46]). Scores on the AAQ-II range from 7 to 49, with higher scores indicating less psychological flexibility. Evidence has been provided for the test-retest reliability and concurrent, predictive, and discriminant validity of this measure [46]. The AAQ-II was administered at pre-treatment, post-treatment, and 3- and 6-month follow-up. Internal consistency in the present sample was excellent (α = .90).

##### Clinician-rated outcome measure

Global functioning was assessed using the Children’s Global Assessment Scale (CGAS [47]). Scores on this measure range from 0 to 100, with higher scores indicating better functioning. Evidence for moderate to excellent inter-rater reliability, good stability over time, and good concurrent and discriminant validity has been provided [47, 48]. The CGAS was administered at pre-treatment, post-treatment, and 3- and 6-month follow-up.

#### Parent-rated outcome measures

Parents’ perceived ability to cope with and respond effectively to their children’s negative emotions was assessed with the Coping with Children’s Negative Emotions Scale – Adolescent version (CCNES-A [49]). Parents rate 36 items on a seven-point Likert type scale ranging from 1 (very unlikely) to 7 (very likely). The CCNES-A version used in this study consists of nine hypothetical scenarios accompanied by four types of responses. The responses assessed for each scenario include distress reactions, punitive reactions, and minimization reactions (for which lower scores indicate more effective responses) and expressive encouragement (for which higher scores indicate more effective responses). The CCNES has demonstrated good test-retest reliability and construct validity [50]. Internal consistency in this sample was adequate for each dimension (αs = .68–.80).

### Treatment

ERITA is an individual therapy for adolescents that was adapted from the original ERGT manual used in several trials of adult women with NSSI [17, 18, 51, 52]. The utility of ERITA as a face-to-face treatment was examined in a previous study, and a detailed description of the treatment is available in that article [16]. In the present study, some modifications were made to both the adolescent treatment and the parent program in response to therapist and participant feedback from the previous study. Specifically, modifications to the adolescent treatment included: (1) combining the four sessions on emotional acceptance, awareness, and clarity into two modules; (2) combining the two sessions on emotional unwillingness and willingness into one module; (3) moving information on valued directions and actions to the first module (vs. the end of treatment) to provide a rationale for approaching emotions early in treatment; and (4) incorporating a greater emphasis on the practice of emotional approach strategies throughout treatment. Table 2 provides an overview of the structure and specific topics addressed in the 11 treatment modules. The modifications to the parent program aimed to increase the emphasis on emotional awareness and validation by replacing the conflict management and problem solving session with one module on the function and awareness of emotions and two modules on validation and invalidation [53]; An outline of the structure and topics addressed in the parent program is available in Table 2.

**Table 2:** An overview of the content of the online ERITA

Adolescent treatment (Module) Content	Parent program (Module) Content
(1) Functions of NSSI and valued directions	(1) Psychoeducation
(2) Impulse control	(2) Emotional awareness
(3) Functionality of emotions and emotional awareness	(3) Validation and invalidation
(4) Primary vs. secondary emotions	(4) Self-validation and self- invalidation
(5) Emotional avoidance / unwillingness vs. emotional acceptance / willingness	(5) How to improve parenting in the long run / behavioral activation
(6) Emotional willingness / approach	(6) Summary and evaluation
(7) Emotional willingness / approach	
(8) Non-avoidant emotion regulation strategies	
(9) Validation and emotional approach	
(10) Repetition	
(11) Relapse prevention	

The treatment platform was completely web-based and designed with age-appropriate appearance, animations, and interactive scripts (see Additional file 1: Figure A.1–3). It has previously been tested in several online treatments for a range of mental and medical disorders (e.g., [54–56]). The treatment includes educational texts, animated films, illustrations, case examples, and interactive exercises. For this study, a mobile app was developed to complement the adolescent online treatment. This app provided the adolescents with the opportunity to report on both self-destructive behaviors and impulses to engage in these behaviors on a daily basis. If the participant had neither engaged in a self-destructive behavior nor experienced impulses to do so, they were instructed to report on potential protective factors. Furthermore, the mobile app (see Additional file 1: Figure A. 4) included reminders of homework assignments and several built-in programs providing assistance in the skills acquired in the online treatment (e.g., impulse control strategies, emotional awareness and clarity, and distraction and approach strategies). Moreover, the individualized crisis plan (see “Participant safety”) could easily be accessed through the app.

During the 12-week treatment period, both adolescents and parents had online contact with an assigned clinical psychologist. The adolescents completed one module every week (i.e., they had one extra week to complete the 11 modules) and the parents completed a module every other week. The psychologist reviewed the participants’ responses and provided written feedback through the platform. In the case of participant inactivity, the psychologist reminded the participant through text messages or telephone calls. Further, the psychologist helped problem solve, guide participants through the program, and assist with the homework assignments when necessary. The amount of therapist time spent with each adolescent and parent was measured.

#### Participant safety

In order to ensure participant safety and facilitate detection of sudden deterioration, participants were instructed to complete online weekly assessments of NSSI and suicidal ideation. An individualized crisis plan was established prior to treatment start, which contained necessary contact information to acute health care services. If increased suicidality was observed, the adolescent and/or parent were immediately contacted.

### Statistical analysis

Statistical analyses were conducted using R version 3.3.1 [57]. Generalized estimation equations (GEE) with exchangeable working correlation structure along with robust error estimations were used to model change in outcome measures across the treatment and follow up periods (from pre-treatment to post-treatment to 3- and 6-month follow-up), as well as for the weekly measures administered before and during treatment. The GEE models for the count variables (i.e., NSSI frequency and self-destructive behaviors) used a negative binomial distribution with a log link function, and the remaining outcomes were analyzed using GEE models with a normal distribution.

Consistent with the use of an intent-to-treat sample, all models included all available data for each outcome. We estimated separate coefficients for the change from pre- to post-treatment, post-treatment to 3-month follow-up, 3-month follow-up to 6-month follow-up, and pre-treatment to 6-month follow-up. We included regression weights in the GEE models that were inversely related to the probability of a value being observed as a function of time (which gives unbiased estimation under the assumption of data missing at random [58, 59]. Ordinal variables were analyzed using Wilcoxon signed ranks test. Effect sizes were calculated for changes between all assessment points. The average percentage change across time for the count variables (i.e., NSSI frequency and BSL) was calculated from the GEE models with 95% confidence intervals. The effect size Cohen’s *d* was calculated for the remaining continuous outcomes by dividing the estimated means derived from the GEE models by the baseline standard deviation. For comparative reasons (with previous studies), Cohen’s *d* was also calculated for the count data based on log-transformed NSSI frequency and BSL scores (these analyses were used only for effect size estimation, and not to draw inferences about statistical significance or percentage change). The 95% confidence intervals for effect sizes were calculated using 5000 bootstrap replications [60] clustered on participants [61]. Finally, we calculated the number of participants who reported no (zero) NSSI episodes at pre-treatment, post-treatment, and 3- and 6-month follow-up and used McNemar’s mid-*p* test [62] to analyze the changes between the assessment points (list-wise deletion was used in these specific analyses when data were missing).

We also examined change in emotion regulation difficulties as a mediator of improvements in NSSI and self-destructive behaviors during treatment in two sets of mediation analyses (with NSSI and self-destructive behaviors coded as the dependent variable in the first and second analysis, respectively). In both sets, two regression equations were estimated [63]. First, the relationship between the independent variable (i.e., time in treatment) and the dependent variable (i.e., *c-path*) was examined to see if time in treatment was associated with frequency of NSSI or self-destructive behaviors. Second, the association between the independent variable and the mediator (i.e., *a-path*) was investigated to test if emotion regulation difficulties decreased as a function of week in treatment. Second, the relationship between the mediator and the dependent variable (i.e., *b-path*) was examined to see if emotion regulation difficulties were associated with NSSI or self-destructive behavior frequency (controlling for time in treatment). The first and third regression equations used a negative binomial distribution with a log link function [64], and the second equation used a normal distribution. The indirect relations of time in treatment to improvements in the dependent variables through improvements in emotion regulation difficulties were calculated as the product of the *a-* and *b-path* estimates, denoted *ab*. A 95% confidence interval (CI) was obtained by 5000 bootstrap replications using the bias-corrected and accelerated method around the *ab*-product, and the criterion for mediation was that the CI for *ab* did not contain zero [65].

## Results

### Feasibility: Treatment completion rates and attrition

Of the 25 participants who started treatment, only one (4%) dropped out of treatment (after the second module, see Fig. 1). The average number of completed treatment modules was 9.7 (*SD* = 2.1; median = 11) out of 11 modules for all included adolescent participants, and 5.2 (*SD* = 1.6; median = 6) out of six modules for the parents. All enrolled adolescents and 24 parents (96%) completed at least one follow-up assessment (see Fig. 1). Mean therapist time for the whole treatment (including reading patient exercises and written feedback) was 309.6 min (*SD* = 133.1) per family (the adolescent and parent), with an average of 238.5 (*SD* = 115.4) and 93.9 (*SD* = 44.2) minutes for the adolescents and parents, respectively. The therapist had additional contact outside of the platform with five of the participants (20%) and their parents through phone calls (*M* = 37 min; *SD* = 13.6 min). At post-treatment, ten participants (40%) reported having some form of treatment contact (e.g., a social worker, psychologist, or medical doctor) while engaged in the online ERITA. However, five of these reported that they had only met with their treatment provider once or twice a month (with the remaining five reporting they had met with their treatment provider once a week during the course of treatment).

### Acceptability: Treatment satisfaction, credibility, and adverse events

Mean ratings of treatment credibility (*M* = 6.5, *SD* = 1.1) and expectancy (*M* = 55.6%, *SD* = 21.4) completed after the first session were satisfactory and very similar to the face to face ERITA trial [16]. In general, participants also rated their alliance with their therapist as very high (*M* = 59.8, *SD* = 15.2). At post-treatment, mean ratings of treatment satisfaction were acceptable for both adolescents (*M* = 17.3, *SD* = 4.6) and parents (*M* = 17.8; *SD* = 3.6).

Eight adolescents reported experiencing one or more adverse events during the treatment, including increased distress (*n* = 7), lack of time for school homework and other obligations (*n* = 3), and feeling depressed when having difficulties practicing the treatment skills (*n* = 1).

### Utility: Outcome analyses

Medians, inter quartile range, means, standard deviations, percentage change, and Cohen’s *d* for the adolescent outcomes are presented in Table 3 and for the parent outcomes in Table 4.

**Table 3:** Adolescent treatment outcome variables at pre-treatment, post-treatment, 3- and 6-month follow-up

Outcome	Pre-treatment	Post-treatment	3-mo f-u	6-mo f-u	Pre- to post-treatment		Post- to 3-mo f-u		3-mo f-u- to 6-mo f-u		Pre to to 6-mo f-u	
Count- data	Median (IQR)	Median (IQR)	Median (IQR)	Median (IQR)	Z	% change [95% CI]	Z	% change [95% CI]	Z	% change [95% CI]	Z	% change [95% CI]
DSHI-9-f	9 (3–15)	2 (0–8)	0 (0–3)	1 (0–6)	3.43^***^	55 [29–72]	3.34^***^	52 [26–69]	−1.35	−44 [− 144–15]	3.95^***^	69 [45–83]
Continuous	Mean (SD)	Mean (SD)	Mean (SD	Mean (SD)	Z	Cohen’s d [95% CI]	Z	Cohen’s d [95% CI]	Z	Cohen’s d [95% CI]	Z	Cohen’s d [95% CI]
DSHI-9-v	2.2 (1.2)	1.4 (1.3)	0.8 (1.0)	0.9 (1.0)	2.33^*^	0.63 [0.54,0.77]	2.97^**^	0.54 [0.47,0.66]	−0.36	−0.06 [−0.5,-0.08]	4.73^***^	1.11 [0.96,1.35]
DERS	125.6 (20.1)	110.6 (27.2)	101.3 (23.3)	99.7 (26.8)	3.14^**^	0.75 [0.59,0.90]	2.07^*^	0.39 [0.31,0.47]	0.34	0.06 [0.05,-0.08]	4.40^***^	1.20 [0.95,1.44]
AAQ	31.4 (9.3)	29.0 (9.8)	27.8 (7.8)	27.6 (9.3)	2.28^*^	0.27 [0.22,0.31]	0.42	0.06 [0.05,0.07]	0.21	0.03 [0.03,-0.04]	2.07*	0.36 [0.30,0.43]
BPFSC	67.1 (12.9)	67.3 (13.9)	65.0 (14.1)	64.4 (12.2)	0.16	0.02 [0.02,0.03]	1.73	0.18 [0.15,0.20]	0.23	0.03 [0.02,-0.04]	1.51	0.23 [0.19,0.27]
CGAS	52.4 (7.1)	60.0 (9.8)	64.6 (8.3)	63.2 (10.6)	3.79^***^	1.01 [0.77,1.32]	3.76^***^	0.53 [0.40,0.70]	0.51	−0.10 [−0.14,-0.08]	4.59^***^	1.44 [1.09,1.88]

*Note.* Count data. Test statistics are based on generalized estimation equation models using either a negative binomial or normal distribution for count and continuous data, respectively. Confidence intervals for effect sizes are based on 5000 bootstrap replications

*Abbreviations*: *AAQ* acceptance and action questionnaire, *BPFSC* borderline personality feature scale for children, *CGAS* Children’s Global Assessment Scale, *DERS* difficulties in emotion regulation scale, *DSHI-9-f* deliberate self-harm inventory – frequency past month, *DSHI-9-v* deliberate self-harm inventory – versatility past month

^*^p < .05; ^**^p < .01; ^***^p < .001

**Table 4:** Parent treatment outcome variables at pre-treatment, post-treatment, 3- and 6-month follow-up

Outcome	Pre-treatment	Post-treatment	3-mo f-u	6-mo f-u	Pre- to post-treatment		Post- to 3-mo f-u		3-mo f-u- to 6-mo f-u		Pre to 6-mo f-u	
Continuous	Mean (SD)	Mean (SD)	Mean (SD	Mean (SD)	Z	Cohen’s d [95% CI]	Z	Cohen’s d [95% CI]	Z	Cohen’s d [95% CI]	Z	Cohen’s d [95% CI]
CCNES-DR	2.0 (0.8)	1.9 (0.8)	1.9 (1.0)	1.9 (1.1)	0.54	0.11 [0.09,0.15]	−0.36	−0.05 [− 0.6,-0.04]	0.30	0.09 [0.07,0.11]	0.54	0.15 [0.12,0.19]
CCNES-PR	1.5 (0.5)	1.3 (0.3)	1.1 (0.2)	1.3 (0.4)	2.27^*^	0.47 [0.35,0.60]	3.31^***^	0.34 [0.25,0.43]	−1.84	−0.26 [0.20,0.34]	4.37^***^	0.55 [0.41,0.70]
CCNES-EE	5.4 (0.9)	5.9 (0.7)	5.8 (1.1)	5.8 (1.0)	3.31^***^	0.58 [0.49,0.68]	−0.46	−0.10 [0.08,0.11]	0.00	0.0 [0.00,0.00]	2.81^**^	0.48 [0.41,0.57]
CCNES-MR	2.9 (0.9)	1.8 (0.6)	1.6 (0.6)	1.7 (0.6)	6.54^***^	1.22 [0.99,1.44]	2.03^*^	0.21 [0.17,0.25]	−0.24	−0.05 [0.01,0.02]	9.08^***^	1.39 [1.15,1.67]

Note. Test statistics are based on generalized estimation equation models using normal distribution. Confidence intervals for effect sizes are based on 5000 bootstrap replications

*Abbreviations*: *EE* expressive encouragement, *DR* distress reactions, *MR* minimization reactions, *PR* punitive reactions

^*^p < .05; ^**^p < .01; ^***^p < .001

#### Primary outcomes

There was a significant 55% reduction in NSSI frequency from pre-treatment to post-treatment. Additionally, results revealed a further 52% significant reduction from post-treatment to 3-month follow-up, which was maintained at 6-month follow-up. The overall change in NSSI frequency between pre-treatment and 6-month follow-up was associated with a significant 69% reduction. The observed means for NSSI frequency were 10.0 (*SD* = 9.3), 4.5 (*SD* = 4.8), 2.2 (*SD* = 3.2), and 3.1 (*SD* = 4.7) at pre-treatment, post-treatment, and 3- and 6-month follow-ups, respectively. Effect sizes (Cohen’s *d*) based on log-transformed data on past month NSSI frequency showed a large reduction from pre- to post-treatment (*d* = 0.88, 95% CI: 0.73, 1.06), a medium reduction from post-treatment to 3-month follow-up (*d* = 0.57, 95% CI: 0.46, 0.68), and no change from 3- to 6-month follow-up (*d* = − 0.09, 95% CI: -0.10, − 0.07). Finally, between pre-treatment and 6-month follow-up, the overall change in NSSI frequency was associated with a large effect size (*d =* 1.36, 95% CI: 1.12, 1.63). The GEE models revealed a significant, medium-sized reduction in past-month NSSI versatility from pre- to post-treatment, with additional significant improvements in NSSI versatility from post-treatment to 3-month follow-up, and no significant changes from 3- to 6-month follow-up. Finally, the proportion of participants with past month NSSI abstinence increased significantly (*p* = .007) from 0% at pre-treatment to 28% at post-treatment, and remained stable from post-treatment to 3- and 6-month follow-ups (48 and 40%, respectively).

#### Secondary outcomes

Results revealed significant medium-sized improvements in emotion regulation difficulties (on the DERS) and large improvements in global functioning (on the CGAS) from pre- to post-treatment, both of which were further improved upon at 3-month follow up and maintained at 6-month follow-up. Likewise, results revealed significant improvements (associated with a small effect size) in psychological flexibility on the AAQ from pre- to post-treatment, which were maintained throughout the follow up period. Notably, however, BPD symptoms did not change significantly during treatment or follow-up.

#### Weekly measures

**Fig. 2:**
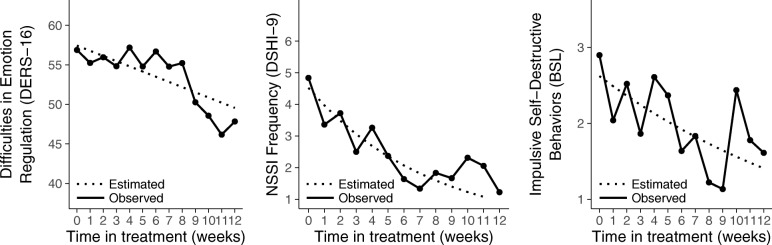
Observed means and estimated regression lines show a significant decrease in difficulties in emotion regulation (*p* = .001), NSSI frequency (*p* < .001), and other self-destructive behavior (*p* = .012) during the course of treatment

Difficulties in emotion regulation (on the DERS-16) decreased significantly throughout treatment (*d =* 0.62; 95% CI: 0.51, 0.75; *p* = .002). Similarly, the GEE analyses revealed that NSSI frequency (on the DSHI-9) and self-destructive behaviors (on the BSL) decreased significantly across treatment, with estimated mean week-to-week reductions of 10.1% (95% CI: 3.8, 15.1; *p* = .001) and 5.2% (95% CI: 1.1, 8.8; *p* = .01), respectively (see Fig. 2).

#### Parent outcomes

Parents’ distress reactions to their children’s expression of negative emotions (on the CNNES-DR) did not improve during treatment or follow-up. However, parental punitive reactions to their children’s expressions of negative emotions (on the CNNES-PR) decreased significantly from pre- to post-treatment and were further improved upon during follow-up. Parental support and encouragement of children’s emotional expressions (on the CCNES-EE) also improved during the treatment period, and these improvements were maintained at the 3- and 6-month follow-ups. Finally, parental minimization of children’s negative emotions (on the CCNES-MR) also improved significantly from pre- to post-treatment, with further improvements in this outcome from post-treatment to 3-month follow up (and maintenance of these gains at the 6-month follow-up).

#### Mediation analysis

The relations between time in treatment and improvements in both NSSI frequency (*c-path* = − 0.101; 95% CI: -0.161, − 0.046) and emotion regulation difficulties (*a-path* = − 0.657; 95% CI: -1.056, − 0.278) were significant, as was the association between emotion regulation difficulties and NSSI improvements during treatment, controlling for time in treatment (*b-path* = 0.029; 95% CI: 0.005, 0.052). Likewise, the indirect relation of time in treatment to NSSI improvement through change in emotion regulation difficulties was significant (*ab* = − 0.019; 95% CI: - 0.045, − 0.003). The corresponding GEE analyses for self-destructive behaviors showed a significant *c-path* (− 0.052; 95% CI: -0.086, − 0.008), *a-path* (− 0.657; 95% CI: -1.056, − 0.278), and a near significant *b-path* (0.016; 95% CI: -0.004, 0.031). Finally, the indirect relation of time in treatment to improvement in self-destructive behaviors through change in emotion regulation difficulties was also significant (*ab* = 0.011; 95% CI: 0.025, 0.001).

## Discussion

NSSID is common among adolescents and associated with a wide variety of negative outcomes. However, treatments developed specifically for NSSI are scarce, and access to empirically-supported treatments for this behavior is limited [9]. Online treatments carry several advantages compared to traditional face-to-face psychological treatments and may be particularly useful for treating NSSI, as this behavior is associated with high levels of shame and non-disclosure [23, 24]. Support for the feasibility and acceptability of online ERITA was provided by findings of high levels of treatment module completion and low treatment attrition, acceptable ratings of treatment credibility, expectancy, and satisfaction (by adolescents and their parents), as well as strong ratings of therapeutic alliance. Providing initial support for the utility of this online treatment for NSSI, participation in the study was associated with significant, medium- to large-sized improvements in past-month NSSI frequency and versatility, emotion regulation difficulties, and global functioning from pre- to post-treatment. Moreover, all of these improvements were either maintained or further improved upon at 3- and 6-month follow-ups. Not only are these findings promising and suggestive of the utility of this online treatment, they are comparable to the findings obtained in previous trials of face-to-face versions of ERITA and ERGT [16–18, 51, 52]. Notably, however, the mean therapist time per family in the current study was approximately one third of the time required in brief face-to-face treatments for NSSI (e.g., [16, 66]) and this substantial reduction in therapist time was managed without a related loss in the feasibility, acceptance, or utility of the intervention. Moreover, consistent with past research on ICBT for adults [67], the therapeutic alliance was strong and comparable to the ratings obtained in the face-to-face trial of ERITA [16]. This is notable given that most therapist support was only provided online; although telephone contact was allowed between therapists and participants, this option was not used for most participants. Even so, participant enrollment in the treatment was associated with durable improvements in behavioral problems, emotion regulation difficulties, and global functioning.

The high treatment completion rates and overall positive findings obtained in this study may be due, in part, to some of the advantages of online interventions in general, such as the structured treatment format, lesser impact of therapist drift, lack of a need to schedule appointments, and greater access to the treatment material. It may also reflect the particular utility of the method of communication inherent to online treatment for individuals with stigmatizing behaviors (e.g., secure self-disclosure [68]). In order to further develop and refine online treatments, future research should include qualitative interviews exploring the experience of participating in online treatments for NSSI.

Despite the positive results found in many domains, only small non-significant improvements were found in BPD symptoms at follow-up. These findings suggest that this brief treatment may not be sufficient to address BPD symptoms beyond emotion dysregulation, NSSI, and other self- destructive behaviors, and that this online treatment may be needed to be incorporated into an overall treatment approach for individuals with BPD for long term remission. However, only 20% of the participants in the present sample met diagnostic criteria for BPD at baseline; thus, replication in larger samples, including larger proportions of adolescents with BPD, is needed before any conclusions regarding the treatment’s utility for BPD symptoms can be drawn.

Family support is important in the treatment of adolescents with self-injurious behaviors [69]. Consistent with both expectations and the intended purpose of the parent program, the online ERITA parent program was associated with small- to large-sized improvements in parental punitive and minimizing responses to adolescents’ expressions of negative emotions and parental encouragement of their children’s emotional expressions. Given past findings that parental invalidation is associated with higher levels of adolescent externalizing problem behaviors, and parental validation is associated with lower levels of emotion dysregulation [70], these findings may, at least in part, account for some of the observed improvements in adolescent NSSI and emotion dysregulation. Contrary to expectations, however, no improvements were found for parental distress reactions to children’s negative emotions. These results suggest that behavioral responses to children’s emotions may be more amenable to brief interventions (and easier to change) than emotional reactions, highlighting the utility of teaching parents’ adaptive ways of responding to children’s distress regardless of their own emotional reactions to that distress. Indeed, even with increased knowledge about the function of and motivations for NSSI, it is reasonable to expect that parents still experience strong emotional reactions to the occurrence of NSSI in their child. However, our findings suggest that, even in the context of high levels of emotional distress, parents can respond behaviorally in an effective manner, possibly due to the emotional awareness and validation skills taught in the parent course. Overall, these findings are encouraging and highlight the utility of further research examining the role of changes in parental behaviors and parents’ own emotion regulation skills in treatment outcomes among adolescents with NSSI and other maladaptive behaviors.

Consistent with past research on both ERGT [71, 72] and ERITA [16], results of the present trial provided further support for the mediating role of change in emotion regulation difficulties in NSSI improvements during treatment. Change in emotion regulation difficulties also mediated improvements in self-destructive behaviors during treatment. These findings provide further support for the underlying role of emotion regulation difficulties in the maintenance of self-destructive behaviors, as well as for emotion regulation as a key mechanism of change in ERGT-based treatments.

Several limitations warrant mention. First, the absence of a control group and/or randomized controlled design precludes any conclusions regarding the effects of this treatment versus the passage of time or other factors. Likewise, ten participants (40%) reported having some form of face to face treatment contact while engaged in the online ERITA. However, it is important to note that five of these reported minimal contact with this treatment provider over the course of treatment. Thus, it is unlikely that the observed improvements in this trial were the result of these additional treatment contacts alone. Nonetheless, further research examining the effects of this treatment in a randomized controlled trial is needed. Second, the majority of participants were self-referred and highly educated, potentially limiting the generalizability of this sample to more complex or severe patient populations. However, findings that 60% of the adolescents in this sample had a history of psychological treatment and rates of co-occurring psychiatric disorders were high suggest that the sample may be representative of a clinical sample in terms of treatment history and psychological burden. Third, the relatively small sample size limits the generalizability of our findings and reduces our statistical power. Finally, the sample largely consisted of girls, limiting our ability to generalize our results to boys.

## Conclusions

The present study provides preliminary support for the acceptability, feasibility, and utility of an online version of ERITA for adolescents with NSSID and their parents, as well as the theory underlying the treatment. Given the benefits of an online treatment format, particularly with regard to therapist time, patient ease and flexibility of scheduling, access to treatment in underserved areas, and its usefulness for stigmatized behaviors, further research examining the efficacy of online ERITA in a larger-scale randomized controlled trial is needed.

## Additional file

Additional file 1: Screen shots from the online ERITA treatment platform and of the mobile app. (DOCX 708 kb)

## Abbreviations

AAQ: Acceptance and action questionnaire
ADHD: Attention deficit hyperactivity disorder
BPD: Borderline personality disorder
BPFS-C: Borderline personality feature scale for children
BSL: Borderline symptom list
CBT: Cognitive behavior therapy
CGAS: Children’s global assessment scale
CI: Confidence interval
DERS: Difficulties in emotion regulation scale
DR: Distress reactions
DSHI: Deliberate self-harm inventory
DSM: Diagnostic and statistical manual of mental disorders
EE: Expressive encouragement
ERGT: Emotion regulation group therapy
ERITA: Emotion regulation individual therapy
GEE: Generalized estimation equation
ICBT: Internet-delivered cognitive behavior therapy
MR: Minimization reactions
NSSI: Nonsuicidal self-injury
NSSID: Nonsuicidal self-injury disorder
PR: Punitive reactions
