# Supplementary material for: Extending research on Emotion Regulation Individual Therapy for Adolescents (ERITA) with nonsuicidal self-injury disorder: open pilot trial and mediation analysis of a novel online version
Source: BMC Psychiatry. 2018 Oct 11;18:326. doi: 10.1186/s12888-018-1885-6 (PMC6180600; doi:10.1186/s12888-018-1885-6)

*Figure A.1*. Screenshot of an interactive work sheet from online ERITA


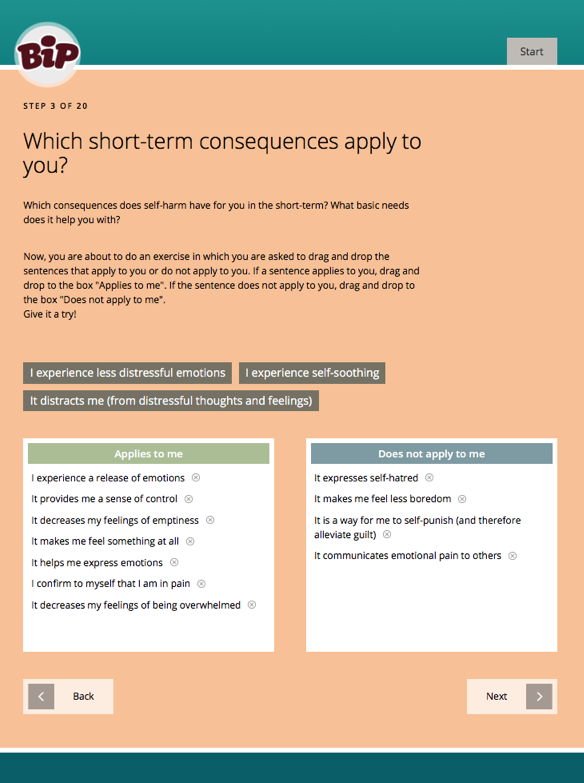


*Figure A.2*. Screenshot of a worksheet with psychoeducative text and illustration from online ERITA


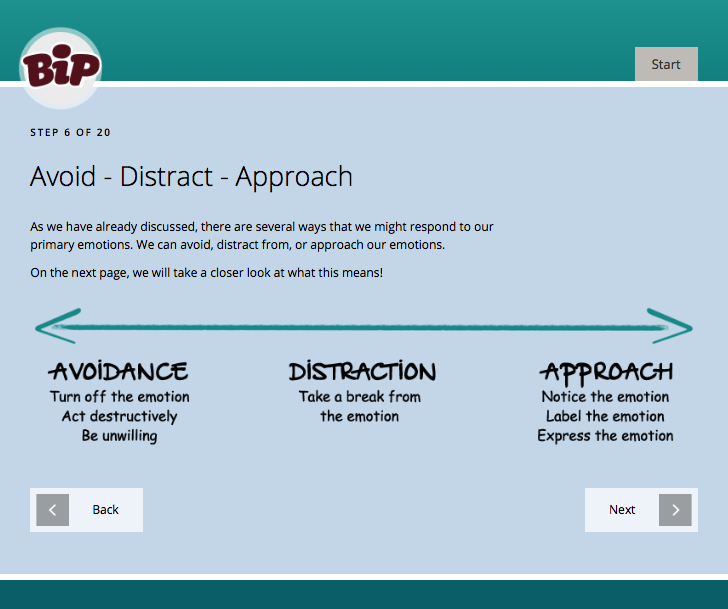


*Figure A.3*. Screenshot of a worksheet with psychoeducative text and illustration from online ERITA


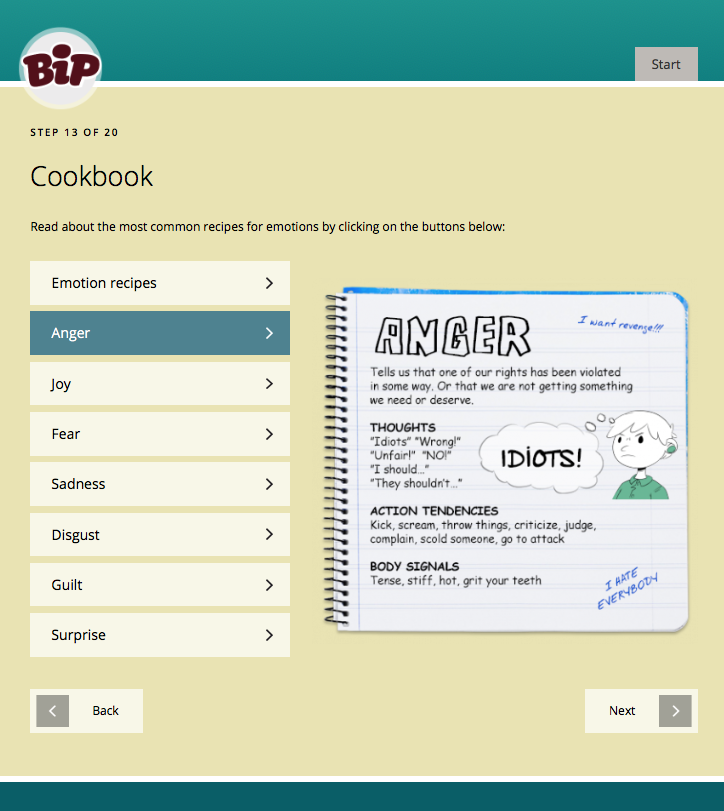


*Figure A.4*. Screen shot of mobile app from online ERITA


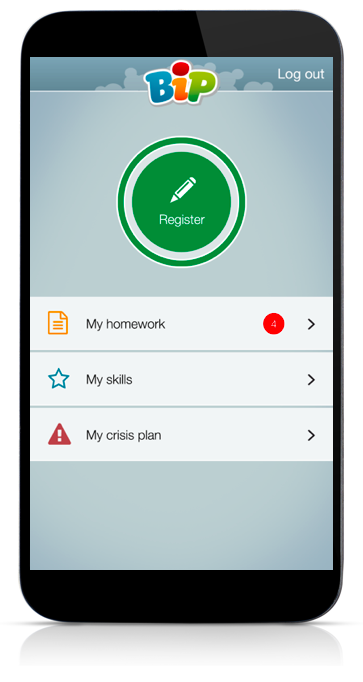

Supplement: Supplementary file 1 — Screen shots from the online ERITA treatment platform and of the mobile app. (DOCX 708 kb) [file 12888_2018_1885_MOESM1_ESM.docx]
